# The effects of supplementing Astragalus and fermented Astragalus on lactation performance, rumen microbiota, and lamb weight gain in Turpan black sheep

**DOI:** 10.3389/fmicb.2026.1810954

**Published:** 2026-05-22

**Authors:** Hao Lu, Guodong Zhao, Tingting Li, Yang Zhou, Tingting Lu, Xiaoying Wei, Jiahao Zhai, Riyilaguli Riyimu, Haibo Lv, Xihu Wang, Jianjun Zhang, Shijie Li, Xiaojun Liu, Rui Xiao, Kailun Yang, Shaohua Zhai

**Affiliations:** 1 Xinjiang Herbivore Nutrition Laboratory for Meat and Milk, College of Animal Science, Xinjiang Agricultural University, Urumqi, China; 2 Xinjiang Hutubi Breeding Cattle Farm Co., Ltd., Changji, China; 3 College of Veterinary Medicine, Xinjiang Agricultural University, Urumgi, China; 4 Xinjiang Key Laboratory of New Drug Research and Development for Herbivorous (XJ-LNDRDH), Urumgi, China; 5 Huishang Ecological Animal Husbandry Co., Ltd., Toksun County, Turpan, China; 6 Xinjiang Animal Embryo Engineering Technology Research Center, Changji, China

**Keywords:** Astragalus, fermented Astragalus, ewes, lambs, immune function

## Abstract

This trial selected Turpan Black ewes at 100 days of gestation as test subjects to investigate the effects of supplemental feeding with Astragalus and fermented Astragalus on the ewes' rumen microbial community structure, plasma metabolites, antioxidant capacity, milk composition, as well as the immune parameters and growth performance of newborn lambs, in order to provide a theoretical basis for stress alleviation and the application of Astragalus and fermented Astragalus. The trial selected 60 Turpan Black ewes at 100 days of gestation and randomly divided them into three groups: Control group (CON group): basal diet; Astragalus group (AM group): basal diet + 2 g/day·head of Astragalus; Fermented Astragalus group (FAM group): basal diet + 2 g/day·head of fermented Astragalus. Each group consisted of 20 ewes, with a 5-day adaptation period and a 90-day experimental period. Dietary supplementation with Astragalus increased milk yield (*p* >0.05) and significantly enhanced antioxidant capacity, as evidenced by elevated catalase (CAT) activity and reduced nitric oxide (NO) levels (*p* < 0.01). Metabolomic analysis revealed that the treatment upregulated picolinic acid and related metabolites, thereby activating the tryptophan metabolic pathway (*p* < 0.01), which consequently improved rumen microbial community structure characterized by increased relative abundances of *Bacteroidetes* and *Firmicutes*. Additionally, membranaceus supplementation elevated birth weight of male lambs by 6.78%, and significantly increased serum concentrations of growth hormone (GH; *p* < 0.01), and immunoglobulins (IgA, IgG, and IgM; *p* < 0.01), accompanied by enhanced antioxidant capacity (*p* < 0.01). Fermented Astragalus membranaceus supplementation increased milk yield and enhanced ewe lactation performance, evidenced by elevated lactose content (*p* < 0.05). The intervention substantially improved systemic redox status, manifesting as increased T-AOC (*p* < 0.01) and augmented antioxidant enzyme activities (elevated CAT, reduced NO and MDA, *p* < 0.01). Rumen microbial profiling revealed significant enrichment of *Bacillales, Methanobacteriales*, and *Spirochaetales* (*p* < 0.05), coupled with metabolomic signatures indicating downregulation of medicarpin and concomitant activation of the α-linolenic acid metabolism pathway (*p* < 0.01). In offspring, the treatment increased male lamb birth weight (*p* < 0.01) and sustained elevation of GH secretion across all developmental stages (*p* < 0.01). Immunologically, neonatal lambs exhibited significantly higher circulating IgA, IgG, and IgM concentrations (*p* < 0.01), alongside enhanced neonatal antioxidant capacity characterized by elevated CAT and reduced NO levels (*p* < 0.01). In summary, fermented Astragalus demonstrated superior intervention effects compared to raw Astragalus in improving ewe milk quality, enhancing antioxidant capacity, optimizing rumen microbial flora structure, regulating lipid metabolism pathways, and promoting lamb growth, immunity, and antioxidant development.

## 1 Introduction

The 45-day prepartum period represents the most critical stage of physiological load in the reproductive cycle of ewes (Chen et al., 2024). During this period, ewes are highly susceptible to various factors, including external environmental stimuli, feeding management conditions, and hormonal fluctuations, which can lead to oxidative stress and decreased immune function, thereby adversely affecting their productive performance as well as the early development and survival of lambs (Zhao J. et al., 2024). Astragalus, a traditional tonic Chinese herbal medicine, contains major active components including Astragalus polysaccharides, saponins, and flavonoids, which play significant roles in enhancing antioxidant capacity, regulating immune function, and promoting nutrient metabolism (Wang and Wu, 2024; Dai et al., 2024). Among these components, astragalosides exhibit significant antioxidant, anti-inflammatory, and immunomodulatory effects. Moreover, the synergistic action of Astragalus polysaccharides and saponins can effectively alleviate oxidative stress in the body (Yao et al., 2023; Hao et al., 2020; Luo et al., 2020). However, conventional Astragalus exists mainly in the form of macromolecular polymers, making the bioactive components inside it, such as saponins, flavonoids, and polysaccharides, difficult to be fully utilized. With the widespread adoption of microbial fermentation technology, the macromolecular substances in Astragalus can be more efficiently released and utilized (Zhou et al., 2024; Niazian and Sabbatini, 2021).

Therefore, proper nutritional management during the prepartum period is not only crucial for ensuring the ewe's health, alleviating physiological stress, and improving ruminal digestion and antioxidant capacity, but it is also of paramount importance for influencing the lamb's birth weight through the placental barrier, providing high-quality colostrum, enhancing the lamb's immunity, and promoting growth performance. This study used 100-day-pregnant Turpan Black ewes as test subjects to investigate the effects of supplemental feeding with Astragalus and fermented Astragalus on the ewes' rumen microbial community structure, plasma metabolites, antioxidant capacity, milk composition, as well as the immune parameters and growth performance of newborn lambs. The findings aim to provide a theoretical basis for alleviating postpartum stress and for the application of Astragalus and fermented Astragalus.

## 2 Materials and methods

### 2.1 Test materials

Astragalus was purchased from the Weishui Hong Cooperative in Gansu; it is in the form of a yellow powder. Fermented Astragalus was purchased from Heilongjiang Lingkang Biotechnology Co., Ltd.: Qiqihar, Heilongjiang, China, it is in the form of a yellow powder, with Bacillus subtilis ≥ 1 × 10^9^ CFU/g and Bacillus licheniformis ≥ 1 × 10^9^ CFU/g.

### 2.2 Test site

The study was conducted from April to July 2025 at Huishang Ecological Pasture Co., Ltd., Toksun County, Turpan City, Xinjiang (coordinates 87°14′05″-89°11′08″E, 41°21′14″-43°18′11″N), spanning 90 days. During this period, the average outdoor temperature was 30.8°C, and the average indoor temperature was 27.5°C.

### 2.3 Trial design and grouping

**Figure 1:**
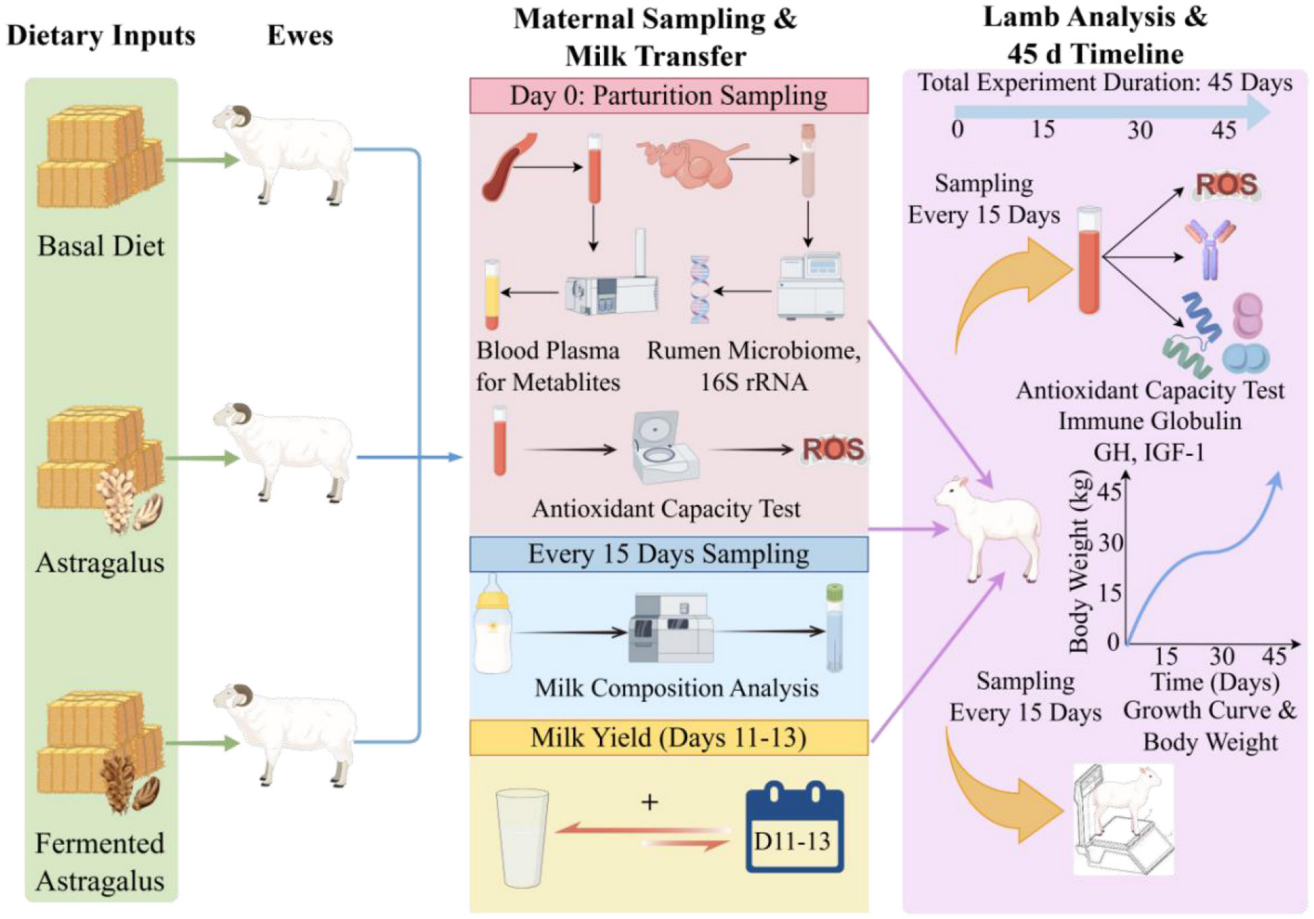
Experimental design flowchart. Basal Diet, CON group; Astragalus, AM group; Fermented Astragalus, FAM group.

Sixty Turpan Black ewes of similar parity and age, selected from the same batch and with synchronized estrus for timed artificial insemination, were randomly divided into three groups of 20 at 100 days of gestation: the control group (CON group), the Astragalus group (AM group), and the fermented Astragalus group (FAM group). The CON group (basic diet + bran), AM group (basal diet + 2 g/day·head of Astragalus), and FAM group (basal diet + 2 g/day·head of fermented Astragalus), until 45 days postpartum. All ewes were reared under identical housing conditions with *ad libitum* feed and water (Figure 1).

### 2.4 Animal husbandry and management

During the trial, all ewes were fed using a TMR (Total Mixed Rations) feeder at 10:30 a.m. and 7:30 p.m. daily, from 45 days before delivery to 45 days after delivery (feed mixture provided by Huishang Ecological Animal Husbandry Co., Ltd., Toksun County, Turpan City, Xinjiang). The CON group received 500 g of bran daily. The AM and FAM group were fed bran mixed with 2 g/d of Astragalus and fermented Astragalus, respectively, and evenly sprinkled into the feed tank. After the ewes had eaten, they were offered a fresh feed supply. The dietary composition and nutritional content of the ewes are shown in Table 1. The lambs born to the ewes in the study had weak or deceased fetuses removed (five per group). The remaining lambs were placed in the same pen as the ewes. Starting at 15 days of age, the lambs were provided with a concentrate supplement (granules), and they had *ad libitum* access to feed and water.

**Table 1:** Diet formula composition and nutrient level (dry matter basis; %).

Ingredients	Content	Nutrient levels	Content
Whole corn silage	35.15	DM②	59.26
Corn husk	35.15	CP	12.87
Concentrate supplement①	15.07	EE	3.08
Sorghum stalks	7.54	Ash	5.54
30-peptide	6.21	NDF	26.54
NaHCO_3_	0.51	ADF	14.03
NaCl	0.31	Ca	0.43
Vitamin D_3_	0.03	P	0.36
Sodium selenite	0.03		

^①^ Premix nutritional levels: DM: 89.46%; CP: 17.00%; EE: 2.28%; Ash: 7.80%; NDF: 40.33%; ADF: 13.92%; Ca: 1.29%; P: 0.54%.

^②^ All nutritional levels were measured.

DM, Dry Matter; CP, Crude Protein; EE, Ether Extract; Ash, Crude Ash; NDF, Neutral Detergent Fiber; ADF, Acid Detergent Fiber; Ca, Calcium; P, Phosphorus.

The lamb concentrate supplement was primarily composed of corn, expanded soybean, soybean meal, corn germ meal, bran, cottonseed protein, mineral elements and their complexes, vitamins and vitaminoids, Saccharomyces cerevisiae (ruminant), amino acids, antioxidants, and other components, all sourced from Xinjiang Jiaruile Biotechnology Co., Ltd.: Changji, Xinjiang, China. The nutrient composition of the lamb concentrate (granules) is listed in Table 2.

**Table 2:** Lamb starter feed (granules; dry matter basis, %)

Items	Nutrient content
CP	20.17
EE	2.15
Ash	9.38
NDF	29.64
ADF	9.24
Ca	2.00
P	0.63

All nutritional levels were measured.

CP, Crude Protein; EE, Ether Extract; Ash, Crude Ash; NDF, Neutral Detergent Fiber; ADF, Acid Detergent Fiber; Ca, Calcium; P, Phosphorus.

### 2.5 Sample collection and preservation

#### 2.5.1 Blood specimen collection

Eight Turpan Black ewes were randomly selected from each group on the day of delivery. Blood was collected from the jugular vein into heparin sodium anticoagulant tubes 1 day before the trial and before morning feeding on the day of delivery. Additionally, blood samples were taken from the jugular veins of lambs born to these ewes at 0, 15, 30, and 45 days postpartum. The blood was centrifuged at 3,500 rpm for 10 min to prepare plasma using a low-temperature high-speed centrifuge (Model 5427R, Eppendorf Life Sciences GmbH, Germany). And the plasma was transferred into 1.8 mL cryopreservation tubes and stored at −80°C after cryopreservation in liquid nitrogen for non-targeted metabolomics, hormone analysis, and antioxidant capacity determination.

#### 2.5.2 Milk sample collection

Milk samples were collected from eight randomly selected Turpan Black ewes in each group via manual milking at 9:00 a.m. and 7:00 p.m. on days 0, 15, 30, and 45 postpartum. Daily milk samples from each ewe were pooled and transferred into 30.0 mL milk sample bottles, which were stored at −20°C for subsequent milk composition analysis.

#### 2.5.3 Feed sample collection

Feed samples were collected using the five-point sampling method combined with the quartering technique. Samples were obtained both before and after lamb feeding. Pre-feeding samples were randomly collected from 5 to 8 points of the fresh daily ration to be offered, with 100 g collected from each point. Post-feeding orts were collected at fixed times after lamb consumption. All collected samples were placed separately into clean trays, thoroughly mixed, and then reduced to a 500 g subsample by quartering. The subsamples were promptly aliquoted, labeled with the corresponding date, and stored for subsequent determination of moisture and nutrient composition.

#### 2.5.4 Rumen fluid collection

Rumen fluid was collected from eight randomly selected ewes from each group on the day of delivery using a sheep gastric tube rumen sampler. Approximately 100 mL of rumen fluid was collected from each ewe, filtered through four layers of gauze, and then divided into 5 mL cryopreservation tubes. The samples were rapidly frozen in liquid nitrogen and stored at −80°C for 16S rRNA sequencing.

### 2.6 Sample analysis

#### 2.6.1 Determination of nutritional levels in diets

Dry matter (DM) content of the diet was determined by the oven drying method at 105°C according to GB/T 6435-2014. Crude ash was determined following GB/T 6438-2025. Crude protein (CP) was analyzed using the Kjeldahl method according to AOAC 990.03. Calcium (Ca) and phosphorus (P) contents were determined by the o-cresolphthalein complexone colorimetric method and the vanadomolybdate colorimetric method, respectively, following AOAC 985.01. Neutral detergent fiber (NDF) and acid detergent fiber (ADF) in the samples were determined according to AOAC 2002.04.

#### 2.6.2 Measurement of lactation yield in ewes

The milk yield of ewes was calculated by measuring the weight difference before and after suckling (An et al., 2022). During the measurement period, all lambs were exclusively fed breast milk. To determine milk yield, eight Turpan Black ewes were randomly selected from each group on the 11th to 13th day postpartum. The lambs were isolated from the ewes at 7:30 a.m., weighed every 3 h, and then allowed to suckle for 10–15 min. After each suckling session, the lambs were immediately isolated and weighed again. The weight difference before and after suckling represented the milk yield for that session. Lactation times were recorded at 10:30 a.m., 2:00 p.m., 5:30 p.m., and 9:00 p.m., and the total milk yield for the day was calculated by summing the differences in weight from all four lactation periods.

#### 2.6.3 Measurement of lamb growth

The birth weight of lambs was recorded, and lambs were weighed before morning feeding every 15 days. Total body weight and average daily body gain (ADG) of the lambs were subsequently calculated.


$$\begin{array}{l} \text{Total Body Weight}=\text{Final Body Weight-Initial Body Weight} \end{array}$$

$$\begin{array}{l} \text{ADG = (Final Weight-Initial Weight)/Trials Days} \end{array}$$

#### 2.6.4 Plasma sample assay

Plasma hormone indicators, including Growth Hormone (GH), Insulin-like Growth Factor 1 (IGF-1), Immunoglobulin A (IgA), Immunoglobulin G (IgG), Immunoglobulin M (IgM), Total Antioxidant Capacity (T-AOC), Catalase (CAT), Superoxide Dismutase (SOD), Glutathione peroxidase (GSH-Px), Malondialdehyde (MDA), and Nitric Oxide (NO), were measured using Enzyme-Linked Immunosorbent Assay (ELISA) kits. The double-antibody sandwich ELISA kits were purchased from Shanghai Yuanju Technology Bio-Center, with the following kit numbers: IGF-1, YJ10602-1; GH, Y7710681-1; IgA, YJ440029-1; IgM, YJ440024; and IgG, YJ440021-1. Antioxidant capacity assays were conducted using kits purchased from Shanghai Enzyme-Linked Bio-Technology Co., Ltd., with the following kit numbers: T-AOC, W96-M(1720); CAT, W96-N(1620); SOD, W96-N(1733); GSH-Px, W96-N(1047); MDA, W96-N(1620); NO, W96-N(1620). Plasma samples collected from experimental ewes on the day of lambing (day 0) were sent to Beijing Novogene Co., Ltd.,: Beijing, China, for differential plasma metabolite analysis.

#### 2.6.5 Determination of milk components

After thawing the milk sample at room temperature, place it in a 40°C water bath for preheating. Following the operating procedures of the automatic milk component analyzer, determine the conventional milk components: milk fat percentage, milk protein percentage, milk lactose percentage, non-fat solids, and ash content. The milk composition analyzer model is MilkoScan™ FT3, purchased from FOSS (Beijing) Technology Trading Co., Ltd.

#### 2.6.6 Rumen fluid analysis

Rumen fluid was collected from experimental ewes on the day of lambing (0 d) and sent to Beijing Novogene Co., Ltd.,: Beijing, China, for 16S rRNA sequencing analysis. Universal bacterial primers (314F:5′-CCTAYGGGRBGCASCAG3′ and 806R:5′-GGACTACNNGGGTATCTAAT3′) were used to amplify the bacterial 16S rRNA V3–V4 region via PCR (polymerase chain reaction). The PCR products were electrophoresed on a 2% agarose gel, purified using magnetic beads, and the target band was recovered for sequencing library preparation. Genomic sequencing was performed on the Illumina NovaSeq platform, and data analysis was conducted using QIIME2 software. Visualization was achieved through the NovoMagic platform.

### 2.7 Data processing and statistical analysis

Data on ewe milk yield, milk composition, plasma antioxidant parameters, and lamb body weight gain were preliminarily organized using Excel. Statistical analyses were then performed using SAS version 9.4 (SAS Institute Inc., Cary, NC, USA). For milk composition, lamb growth performance, plasma hormones, antioxidant indices, and immune parameters, a repeated-measures mixed model was applied. Results are presented as mean ± standard error of the mean (SEM). Ewe milk yield and plasma antioxidant indices on the day of parturition were analyzed by one-way ANOVA using SPSS version 27.0, and differences between groups were assessed by Duncan's multiple range test.

Both plasma metabolite and rumen microbial data were analyzed using the cloud platform provided by Beijing Novogene Biotechnology Co., Ltd. R software version 4.5.1 was used to analyze the correlations between plasma differential metabolites and rumen microbiota as well as milk components. Data visualization was performed using GraphPad Prism 9.5 and Adobe Illustrator 2025. Results are expressed as mean ± standard error. *p* < 0.05 was considered statistically significant, and *p* < 0.01 was considered highly significant.

## 3 Results and analysis

### 3.1 Effects of supplementing with Astragalus and fermented Astragalus on ewes of the Turpan black sheep breed

#### 3.1.1 Effects of supplementing with Astragalus and fermented Astragalus on lactation performance in ewes

##### 3.1.1.1 Effects-of-supplementing-with-astragalus-and-fermented-astragalus-on-milk-yield-in-ewes

As shown in Table 3, supplemental feeding of Astragalus or fermented Astragalus had no significant effect on milk yield in ewes. Compared with the CON group, milk yield in the AM and FAM groups increased by 18.75% and 20.31%, respectively, 11 days postpartum, with no significant differences among the groups (*p* > 0.05); Milk yield in the AM and FAM groups increased by 18.03% and 21.31%, respectively, on day 12 postpartum, with no significant differences among the groups (*p* > 0.05); milk yield in the AM and FAM groups increased by 10.00% and 13.33%, respectively, on day 13 postpartum, with no significant differences among the groups (*p* > 0.05); Total milk yield in the AM and FAM groups from days 11 to 13 increased by 14.52% and 17.20%, respectively, with no significant differences among the groups (*p* > 0.05).

**Table 3:** Effect of supplementing with Astragalus and fermented Astragalus on lactation yield in ewes (*n* = 15, kg).

Postpartum time (d)	Groups			*P*-value
	CON group	AM group	FAM group	
11 d	0.64 ± 0.16	0.76 ± 0.18	0.77 ± 0.20	0.14
12 d	0.61 ± 0.17	0.72 ± 0.19	0.74 ± 0.16	0.13
13 d	0.60 ± 0.19	0.66 ± 0.19	0.68 ± 0.15	0.46
Total Milk Yield	1.86 ± 0.48	2.13 ± 0.50	2.18 ± 0.45	0.14

No significant differences were observed (*p* > 0.05); hence no superscript letters are shown.

##### 3.1.1.2 Effects-of-supplementing-with-astragalus-and-fermented-astragalus-on-the-composition-of-ewes-milk

As shown in Table 4, supplementation with Astragalus and fermented Astragalus increased milk fat content, milk protein content, ash content, and non-fat solids (*p* > 0.05). The lactose content in the Astragalus group and the fermented Astragalus group showed significant differences as the supplementation period increased (*p* < 0.05). Notably, the milk protein content in the fermented astragalus group was significantly higher than that in the astragalus group at day 0 (*p* < 0.05), while the difference compared to the control group was not significant (*p* > 0.05).

**Table 4:** Effects of supplementing with Astragalus and fermented Astragalus on milk composition in ewes (*n* = 6).

Items	Groups			*SEM*	*P*-value		
	CON group	AM group	FAM group		Groups	Time	Groups × time
Fat (%)	8.34	8.74	9.13	0.18	0.06	< 0.01	0.63
Protein (%)	7.89^ab^	7.67^b^	8.20^a^	0.15	0.04	< 0.01	0.07
Lactose (%)	3.94	4.11	4.01	0.11	0.14	< 0.01	0.02
Ash (%)	1.04	1.03	1.11	0.04	0.67	< 0.01	0.66
SNF (%)	12.78	12.69	13.19	0.25	0.10	< 0.01	0.18

Within-group data with no letter or the same lowercase letter indicate no significant difference (*p* > 0.05); different lowercase letters indicate a significant difference (*p* < 0.05); and different uppercase letters indicate a highly significant difference (*p* < 0.01). There was a significant interaction effect between the Protein indicator groups; the letter labels in the same row indicate the results of a simple effects analysis for a specific time point (0d).

#### 3.1.2 Effects of supplementing with Astragalus and fermented Astragalus on antioxidant capacity in ewe plasma

As shown in Table 5, regarding the effects of Astragalus and fermented Astragalus supplementation on the antioxidant capacity of ewe plasma, T-AOC in the FAM group was significantly higher than that in the CON and AM groups (*p* < 0.01); CAT activity in the AM and FAM groups was significantly higher than that in the CON group (*p* < 0.01); there were no significant differences in SOD and GSH-Px among the groups (*p* > 0.05); NO levels in the AM and FAM groups were significantly higher than those in the CON group (*p* < 0.01); MDA levels in the CON and AM groups were significantly higher than those in the FAM group (*p* < 0.01), and MDA levels in the CON group were significantly higher than those in the AM group (*p* < 0.01).

**Table 5:** Effects of supplementing with Astragalus and fermented Astragalus on antioxidant capacity in plasma of ewes on parturition day (*n* = 6).

Items	Groups			*P*-value
	CON group	AM group	FAM group	
T-AOC (U/mL)	4.96 ± 0.96^Bb^	5.06 ± 0.74^Bb^	8.90 ± 0.65^Aa^	< 0.01
CAT (U/mL)	5.60 ± 0.89^Bb^	8.59 ± 1.19^Aa^	9.07 ± 0.89^Aa^	< 0.01
SOD (U/mL)	10.14 ± 1.44	9.94 ± 1.13	10.65 ± 1.26	0.63
GSH-Px (U/mL)	139.89 ± 14.28	142.42 ± 14.09	148.13 ± 7.77	0.52
NO (nmol/mL)	324.16 ± 12.56^Bb^	348.19 ± 8.87^Aa^	345.19 ± 12.02^Aa^	< 0.01
MDA (nmol/mL)	1.46 ± 0.21^Aa^	1.10 ± 0.24^Bb^	0.63 ± 0.20^Cc^	< 0.01

In the data tables, the absence of a letter or the presence of the same letter in the superscript indicates no significant difference (*p* > 0.05); different lowercase letters indicate a significant difference (*p* < 0.05); and different uppercase letters indicate a highly significant difference (*p* < 0.01).

#### 3.1.3 Effects of supplementing with Astragalus and fermented Astragalus on the rumen microbial flora structure of ewes

##### 3.1.3.1 OTU-based Venn diagram analysis

**Figure 2:**
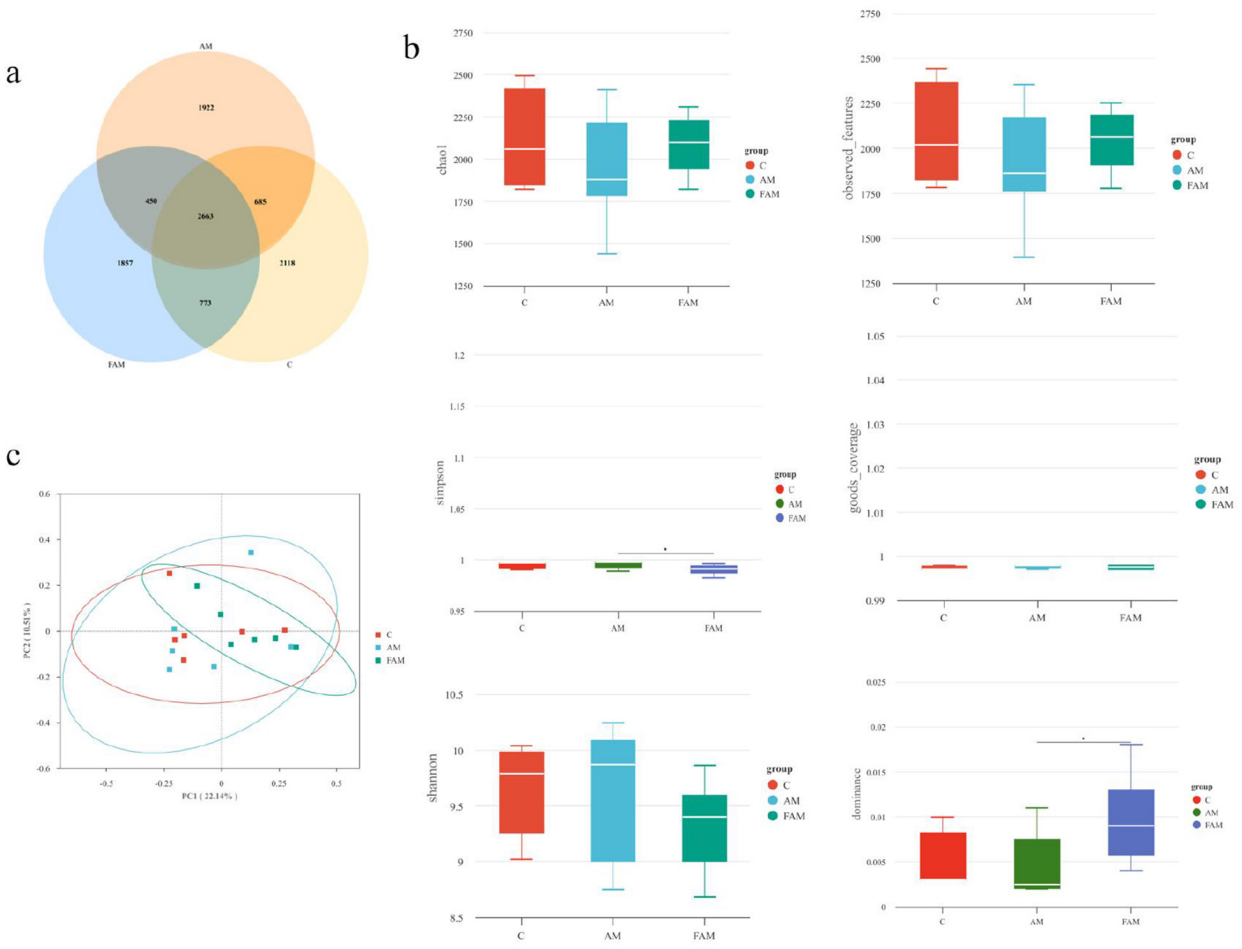
The effects of supplementing with Astragalus and fermented Astragalus on rumen microbial diversity in female Turpan black sheep. **(a)** OTU-based venn diagram analysis; **(b)** analysis of alpha diversity; **(c)** Principal Coordinate Analysis (PCoA) across groups. In this figure, C represents the control group, AM represents the Astragalus group, and FAM represents the fermented Astragalus group. The same applies to the figure below.

The OTU-based Venn diagram analysis of rumen microbiota in the Astragalus and fermented Astragalus supplementation groups is shown in Figure 2a. As shown in the figure, the number of OTUs specific to and shared among the groups is presented. There were 2,663 shared OTUs among the three groups. The control group had 2,118 unique OTUs, the Astragalus group had 1,992 unique OTUs, and the fermented Astragalus group had 1,857 unique OTUs.

##### 3.1.3.2 Analysis of alpha diversity

As shown in Figure 2b, there were no significant differences in the Chao1, coverage, Pielou-e, and Shannon indices among the control group, the Astragalus group, and the fermented Astragalus group (*p* > 0.05). However, the fermented Astragalus group showed a significant difference in the dominance index (*p* < 0.05), indicating that fermented Astragalus has a significant effect on the diversity of the rumen microbial community in ewes. Furthermore, the species coverage of samples in all groups reached 99.9%, suggesting that the sequencing depth accurately reflects the true state of the microbial community in the rumen samples.

##### 3.1.3.3 Principal Coordinate Analysis (PCoA) Across Groups

As shown in Figure 2c, the PCoA plot indicates a clear separation between the groups, demonstrating a significant classification effect. The first principal component explains 22.14% of the variance, while the second principal component explains 10.51%.

##### 3.1.3.4 Effects of supplemental feeding of Astragalus and fermented Astragalus on the rumen microbiota of ewes (phylum level)

**Figure 3:**
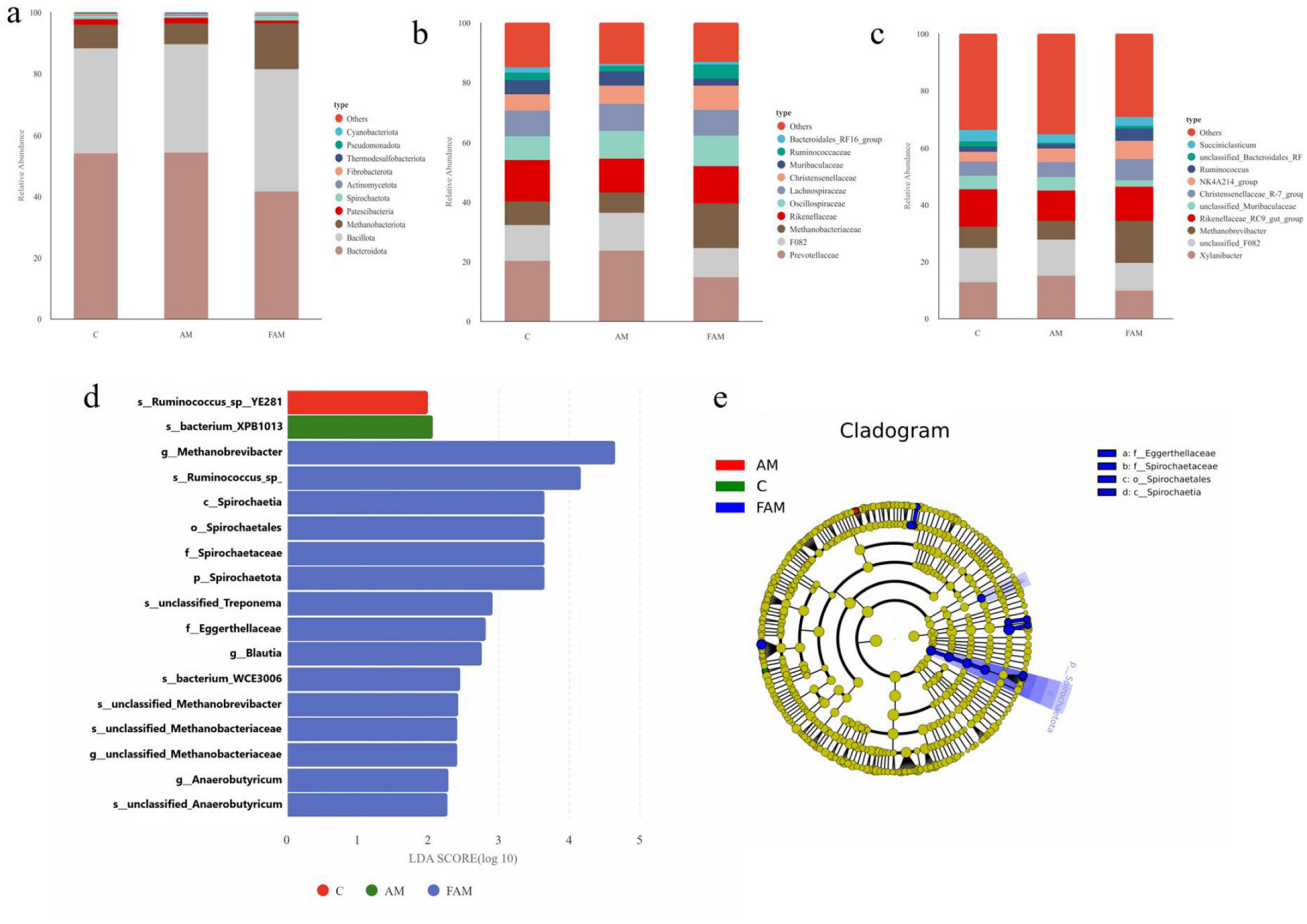
Differential analysis of rumen microbial species composition in ewes supplemented with Astragalus and fermented Astragalus. **(a)** Phylum level; **(b)** Family level; **(c)** Genus level; **(d)** LEfSe analysis; **(e)** Phylogenetic tree. The letters p, c, o, f, g, and s represent the taxonomic ranks of phylum, class, order, family, genus, and species, respectively.

The composition of rumen microbiota at the phylum level in ewes from the CON, AM, and FAM groups is shown in Figure 3a, with the top ten major phyla listed, such as *Bacteroidota* (54.03%; 54.16%; 41.43%), *Bacillota* (34.09%; 35.45%; 39.85%), *Methanobacteriota* (7.76%; 6.79%; 14.99%), *Patescibacteria* (1.86%; 1.83%; 0.91%), *Spirochaetota* (1.12%; 0.67%; 1.60%), *Actinobacteriota* (0.33%; 0.46%; 0.62%), and *Fibrobacterota* (0.36%; 0.14%; 0.28%), *Thermodesulfobacteriota* (0.26%; 0.33%; 0.15%), *Pseudomonadota* (0.10%; 0.05%; 0.06%), and *Cyanobacteria* (0.01%; 0.02%; 0.05%). Among these, the relative abundance of Bacteroidota in CON and AM groups was significantly higher than that in Group FAM (*p* < 0.05), while the relative abundance of Bacillota in Group FAM was higher than that in CON and AM groups. The relative abundance of Methanobacteriota in Group FAM was significantly higher than that in Groups AM and D (*p* < 0.05); the relative abundance of Spirochaetota in the FAM group was higher than that in the AM group (*p* < 0.05) and the CON group (*p* > 0.05; see Appendix A1 for details on statistical significance).

##### 3.1.3.5 Effects of supplementing Astragalus and fermented Astragalus on the rumen microbiota of ewes (family level)

As shown in Figure 3b, the top 10 bacterial families by relative abundance were *Prevotellaceae* (20.21%; 23.54%; 14.68%), *F082* (12.06%; 12.80%; 9.74%), *Methanobacteriaceae* (7.76%; 6.79%; 14.99%), *Rikenellaceae* (13.92%; 11.24%; 12.59%), *Oscillospiraceae* (7.99%; 9.37%; 10.22%), *Lachnospiraceae* (8.51%; 9.02%; 8.57%), *Muribaculaceae* (4.73%; 4.70%; 2.31%), *Christensenelaceae* (5.59%; 6.13%; 8.10%), *Ruminococcaceae* (2.39%; 1.95%; 4.68%), and *Bacteroidales_RF16_group* (1.83%; 0.61%; 0.95%). The relative abundance of *Methanobacteriaceae* in the FAM group was significantly higher than that in the CON and AM groups (*p* < 0.05); the relative abundance of *Ruminococcaceae* was higher than that in the CON group (*p* > 0.05) and the AM group (*p* < 0.05; see Table A2 for details on statistical significance).

##### 3.1.3.6 Effects of supplementing with Astragalus and fermented Astragalus on the rumen microbiota of ewes (genus level)

As shown in Figure 3c, the major annotated bacterial genera include *Xylanibacter* (12.72%; 14.88%; 9.80%), *unclassified_F082* (12.06%; 12.80%; 9.74%), *Methanobrevibacter* (7.39%; 6.65%; 14.69%), *Rikenellaceae_RC9_gut_group* (13.30%; 10.69%; 12.07%), *unclassified_Muribaculaceae* (4.73%; 4.70%; 2.31%), *Christensenellaceae_R-7_group* (4.99%; 5.11%; 7.33%), *NK4A214_group* (3.43%; 4.88%; 6.44%), *Ruminococcus* (1.82%; 1.48%; 4.25%), *unclassified_Bacteroidales_RF16_group* (1.83%; 0.61%; 0.95%), and *Succiniclasticum* (3.89%; 2.95%; 3.19%). *Methanobrevibacter* in the FAM group was significantly higher than in the AM and CON groups (*p* < 0.05), and *Ruminococcus* was significantly higher than in the AM group (*p* < 0.05) (see Table 3 for statistical significance).

##### 3.1.3.7 Analysis of differences in the species composition of the rumen microbiota in ewes fed Astragalus and fermented Astragalus

Based on the above analysis of community structure and species composition, which indicated that supplemental feeding of fermented Astragalus altered the rumen microbial community structure in ewes, we further employed LEfSe analysis and screened for microorganisms with significant differences in relative abundance among the three groups based on effective patterns identified by LDA (LDA > 2, *p* < 0.05). The results are shown in Figures 3d,e.

The relative abundances of the FAM group's *p_Spirochaetota* (LDA = 3.67), *c_Spirochaetia* (LDA = 3.67), *o_Spirochaetales* (LDA = 3.67), and *f_Spirochaetaceae* (LDA = 3.67) were significantly higher than those in the CON and AM groups (*p* < 0.05); *g_Methanobrevibacter* (LDA = 4.64), g_unclassified_Methanobacteriaceae (LDA = 2.43), s_unclassified_Methanobacteriaceae (LDA = 2.43), and *s_unclassified_Methanobrevibacter* (LDA = 2.43) were significantly higher than those in the CON and AM groups (*p* < 0.05); the relative abundances of *g_Anaerobutyrium* (LDA = 2.27) and the *unclassified s_unclassified_Anaerobutyrium* (LDA = 2.25) were significantly higher than those in the CON and AM groups (*p* < 0.05).

In the FAM group, the relative abundances of *s_Ruminococcus_sp_* (LDA = 4.17), *s_unclassified_Treponema* (LDA = 2.89), *s_bacterium_WCE3006* (LDA = 2.43), *g_Blautia* (LDA=2.78), and *f_Eggerthellaceae* (LDA = 2.82) were significantly higher in the FAM group than in the CON and AM groups (*p* < 0.05).

The relative abundance of s_bacterium_XPB1013 (LDA = 2.04) in the AM group was significantly higher than in the CON and FAM groups (*p* < 0.05).

In the CON group, the relative abundance of s_Ruminococcus_sp_YE281 (LDA = 2.0) was significantly higher than that in the AM and FAM groups (*p* < 0.05).

#### 3.1.4 Effects of supplementing with Astragalus and fermented Astragalus on plasma metabolites in ewes

##### 3.1.4.1 Venn diagram analysis of differentially expressed metabolites

**Figure 4:**
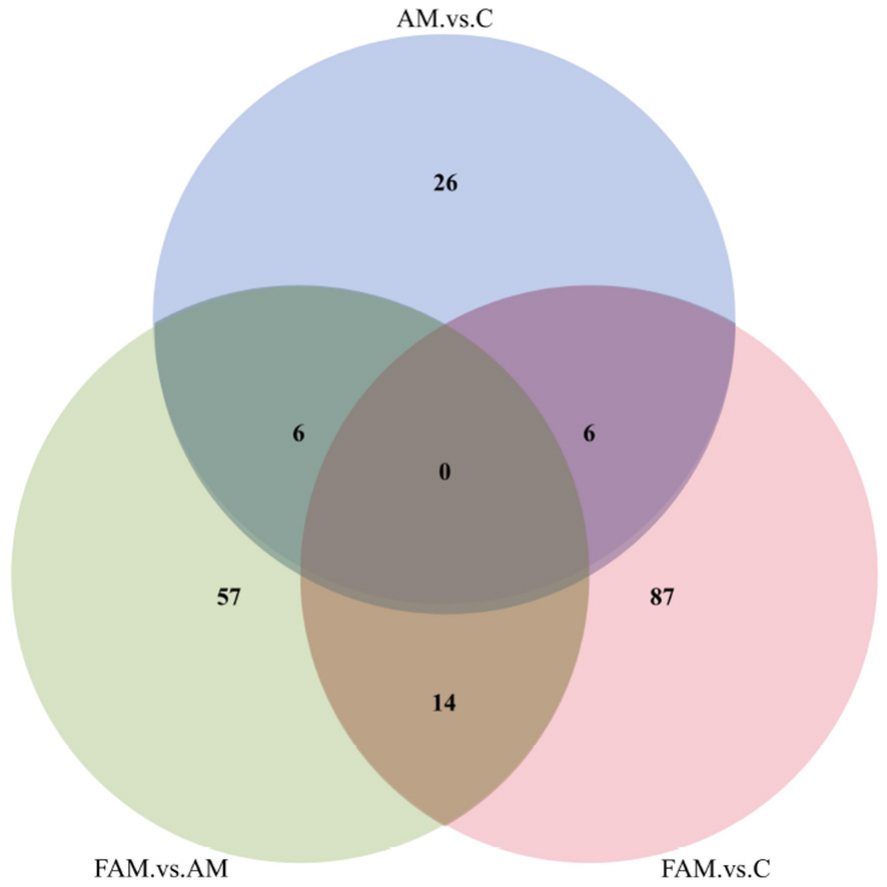
Venn analysis of differential metabolites in plasma from ewes supplemented with Astragalus and fermented Astragalus. In the figure, “AM vs. C” refers to the comparison between the AM group and the CON group; “FAM vs. C” refers to the comparison between the FAM group and the CON group; and “FAM vs. AM” refers to the comparison between the FAM group and the AM group. The same applies to the figure below.

As shown in Figure 4, after processing the sequencing data from the samples, metabolites were classified based on the similarity of their plasma metabolite profiles. A total of 133 distinct metabolites were identified in the comparison pairs between the AM group and the CON group vs. the FAM group and the CON group. Specifically, the comparison pair between the AM group and the CON group contained 38 metabolites, while the comparison pair between the FAM group and the CON group contained 107 metabolites; the two comparison pairs shared six metabolites. The comparison between the AM group and the CON group vs. the comparison between the FAM group and the AM group identified a total of 103 distinct metabolites. Specifically, the comparison between the AM group and the CON group yielded 38 distinct metabolites, while the comparison between the FAM group and the AM group yielded 77 distinct metabolites; the two groups shared 6 common metabolites; The comparison between the FAM group and the AM group, and the comparison between the FAM group and the CON group, identified a total of 156 distinct metabolites. Specifically, the comparison between the FAM group and the AM group identified 77 metabolites, while the comparison between the FAM group and the CON group identified 107 metabolites; the number of metabolites shared between the two comparisons was 14.

##### 3.1.4.2 Partial least squares discriminant analysis (PLS-DA)

**Figure 5:**
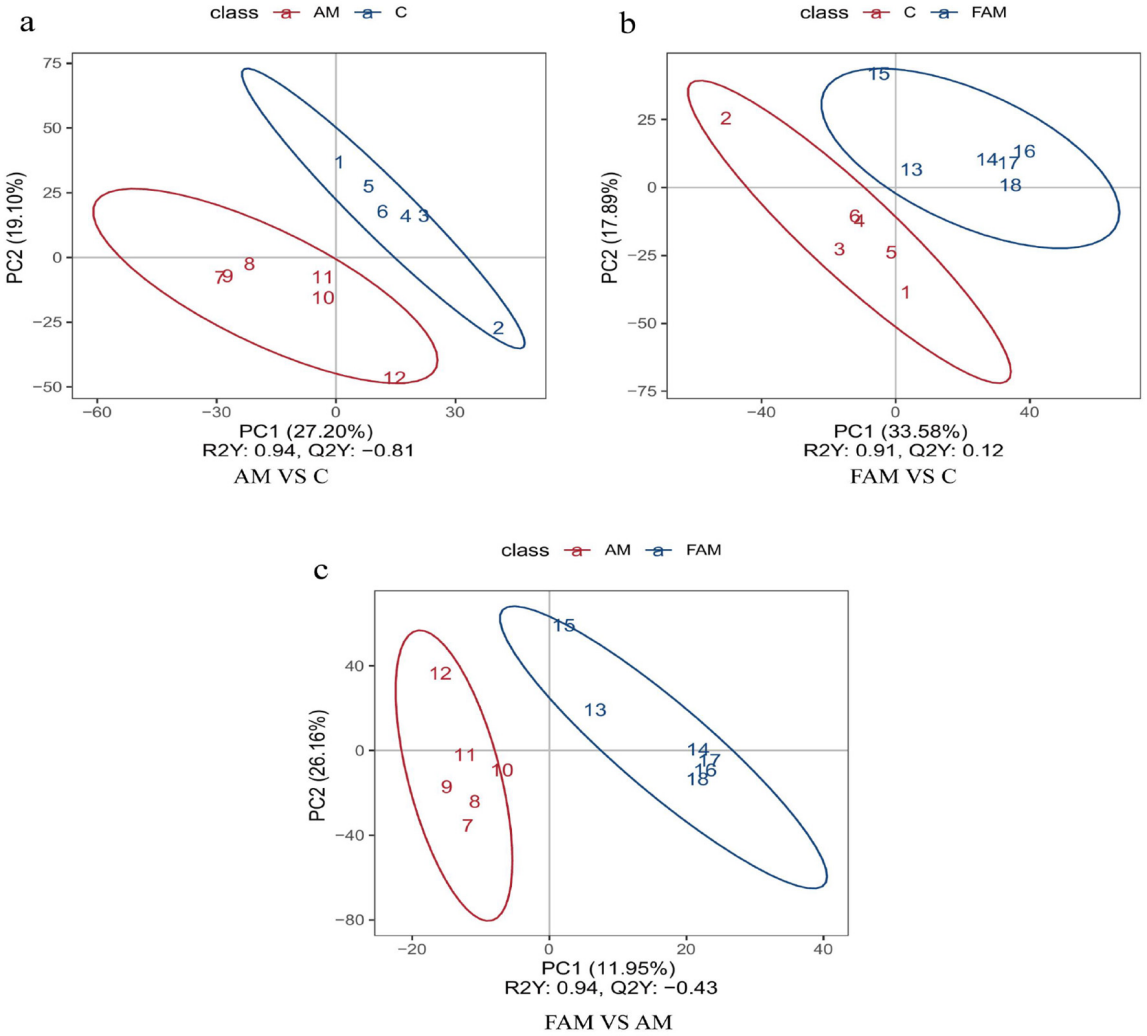
Partial least squares discriminant analysis (PLS-DA) among groups. **(a)** “AM.vs.C” refers to the comparison between the AM group and the CON group; **(b)** “FAM.vs.C” refers to the comparison between the FAM group and the CON group; and **(c)** “FAM.vs.AM” refers to the comparison between the FAM group and the AM group.

As shown in Figure 5, the greater the separation between the two groups of samples in the PLS-DA plot, the more significant the classification performance. The separation between the groups is clear, indicating a relatively significant classification performance.

##### 3.1.4.3 Screening and analysis of metabolites differing between the Astragalus group and the control group

**Figure 6:**
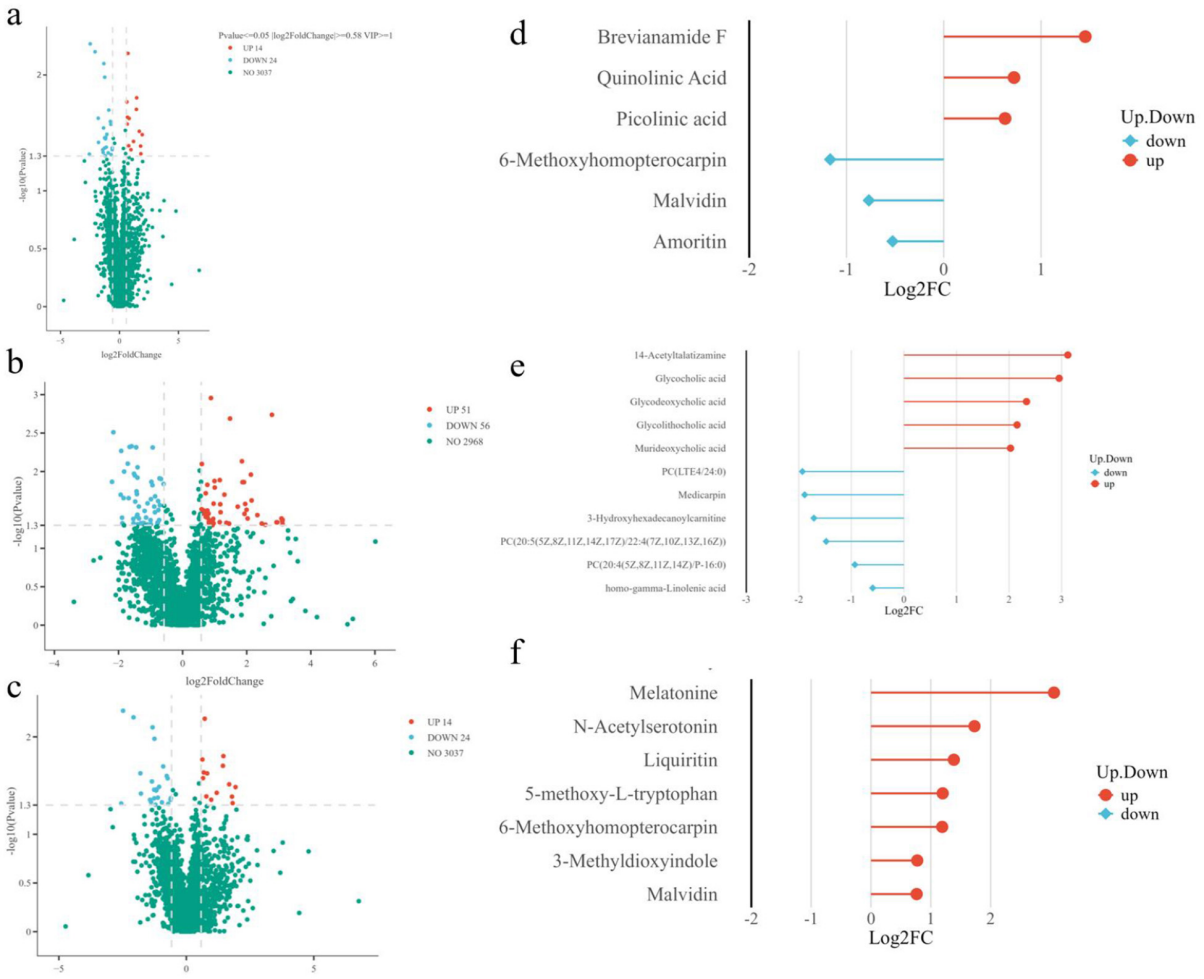
Volcano plot **(a–c)** and bar chart **(d–f)** of differential metabolite screening analysis. Red dots represent metabolites with significantly increased expression, blue dots represent metabolites with significantly decreased expression, and gray dots represent metabolites with no significant differences between groups; the x-axis represents the fold change of metabolites, expressed as log_2_(Fold Change); the closer a point is to either end of the x-axis, the greater the fold change; The vertical axis represents the significance level of the difference, expressed as negative log_10_(*P*-value). The closer a point is to the top of the vertical axis, the smaller the *P*-value, indicating stronger statistical significance and higher reliability of the test; the size of the dot corresponds to the VIP value. A higher VIP value indicates that the metabolite contributes more significantly to the experimental group.

As shown in Figure 6a, a total of 3,075 differentially expressed metabolites were detected in the plasma of ewes in the AM and CON groups. There were 38 significantly altered metabolites, accounting for 1.24% of the total metabolites. Among these, 14 were upregulated (*p* < 0.05), representing 0.46% of the total metabolites and 36.84% of the total differentially expressed metabolites, 24 downregulated differentially expressed metabolites (*p* < 0.05), accounting for 0.78% of the total metabolites and 63.16% of the total differentially expressed metabolites, 3,037 differentially expressed metabolites showed no significant changes (*p* > 0.05). Among the upregulated differentially expressed metabolites, relevant compounds identified included Brevianamide F, Quinolinic Acid, and Picolinic Acid; downregulated differentially expressed metabolites included 6-methoxyhomopterocarpin, malvidin, and amoritin (Figure 6d).

##### 3.1.4.4 Screening and analysis of metabolites differing between the control group and the fermented Astragalus group

As shown in Figure 6b, a total of 3,075 metabolites were detected in the plasma of ewes in the FAM and CON groups. Among these, 107 metabolites showed significant changes, accounting for 3.48% of the total metabolites. Of these, 51 were upregulated metabolites (*p* < 0.05), representing 1.66% of the total metabolites and 47.66% of the total differentially expressed metabolites, 56 downregulated differentially metabolites (*p* < 0.05), accounting for 1.82% of the total metabolites and 52.34% of the total differentially metabolites; 2,968 differentially metabolites showed no significant changes (*p* > 0.05). Among the upregulated differentially expressed metabolites, relevant metabolites identified included 14-Acetyltalatizamine, Glycocholic acid, Glycodeoxycholic acid, and Glycolithocholic acid; Downregulated differentially expressed metabolites included Medicarpin, 3-Hydroxyhexadecanoylcarnitine, and [PC20:5(5Z,8Z,11Z,14Z,17Z)/22:4(7Z,10Z,13Z,16Z)], among others (Figure 6e).

##### 3.1.4.5 Screening and analysis of metabolites differing between the fermented Astragalus group and the Astragalus group

As shown in Figure 6c, a total of 3,075 differentially expressed metabolites were detected in the plasma of ewes in the FAM and AM groups. Among these, 77 metabolites showed significant changes, accounting for 2.50% of the total metabolites. Of these, 49 were upregulated (*p* < 0.05), representing 1.59% of the total metabolites and 63.64% of the total differentially expressed metabolites; 28 downregulated differentially expressed metabolites (*p* < 0.05), accounting for 0.91% of the total metabolites and 36.36% of the total differentially expressed metabolites; 2,998 differentially expressed metabolites showed no significant changes (*p* > 0.05). Among the upregulated differentially expressed metabolites, relevant metabolites identified included melatonin, *N*-acetylserotonin, and malvidin (Figure 6f).

##### 3.1.4.6 KEGG pathway analysis of differentially expressed metabolites

**Figure 7:**
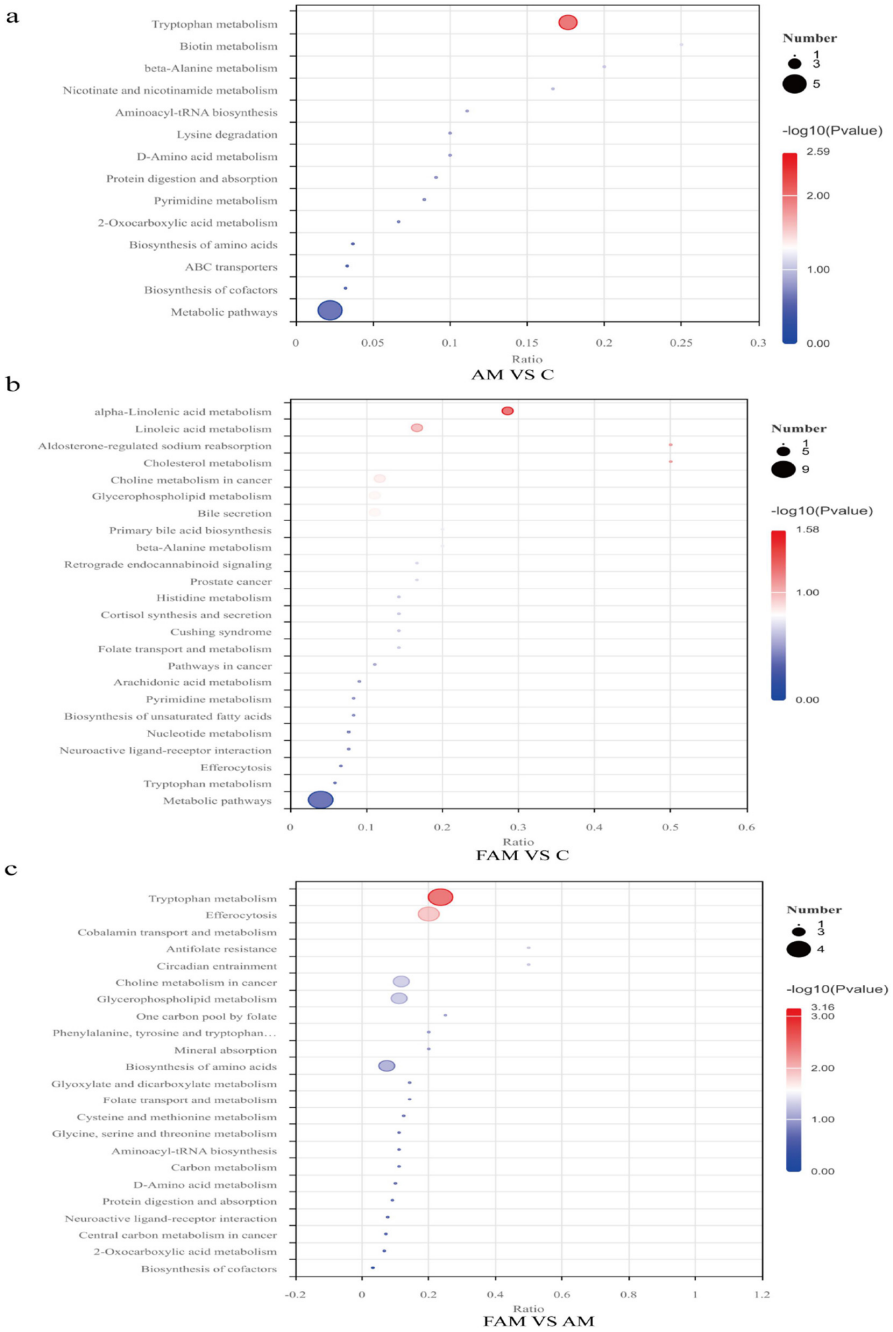
KEGG Enrichment Bubble Plot. The figure displays the top 20 KEGG enriched pathways by *P*-value, sorted from lowest to highest. The *x*-axis represents *x*/*y*; the larger the value, the further to the right the point is located, indicating a higher degree of enrichment of differentially expressed metabolites in that pathway. The color of the point represents the *P*-value from the hypergeometric test; the redder the color, the smaller the *P*-value, indicating greater reliability of the test and more statistically significant results. The size of the point reflects the number of differentially expressed metabolites in the corresponding pathway; the larger the point, the greater the number of differentially expressed metabolites in that pathway. **(a)** “AM.vs.C” refers to the comparison between the AM group and the CON group; **(b)** “FAM.vs.C” refers to the comparison between the FAM group and the CON group; and **(c)** “FAM.vs.AM” refers to the comparison between the FAM group and the AM group.

As shown in Table 6, screening of the KEGG database identified a total of 14 key differentially expressed metabolic pathways (*p* < 0.05) between the AM group and the CON group. The most relevant key differentially expressed metabolic pathway was tryptophan metabolism, as shown in Figure 7a.

**Table 6:** Effects of supplemental feeding with Astragalus and fermented Astragalus on differential plasma metabolites in ewes as identified by KEGG analysis.

ID	Pathway	*P*-value
AM vs. C	Tryptophan metabolism	0.003
FAM vs. C	Alpha-Linolenic acid metabolism	0.03
FAM vs. AM	Tryptophan metabolism	< 0.001
	Efferocytosis	0.007

A total of 24 key differentially expressed metabolic pathways (*p* < 0.05) were identified in the FAM and CON groups; the most relevant key differentially expressed metabolic pathway was alpha-linolenic acid metabolism, as shown in Figure 7b.

A total of 23 key differentially expressed metabolic pathways (*p* < 0.05) were identified in the fermented Astragalus group and the Astragalus group; the most relevant key differentially expressed metabolic pathways were tryptophan metabolism and efferocytosis, as shown in Figure 7c.

### 3.2 Effects of supplementing Turpan black ewes with Astragalus and fermented Astragalus on lambs

#### 3.2.1 Effects of supplementing ewes with Astragalus and fermented Astragalus on lamb body weight

As shown in Table 7, the body weight of male lambs in the Astragalus group and the fermented Astragalus group increased significantly for both treatment and time effects, as well as the interaction between treatment and time (*p* < 0.01). In the fermented astragalus group, daily weight gain in male lambs, body weight in female lambs, and daily weight gain in female lambs showed highly significant differences for both group and time effects (*p* < 0.01), while there were no significant differences for the group-time interaction effect (*p* > 0.05).

**Table 7:** Effect of supplementing ewes with Astragalus and fermented Astragalus on lamb body weight (*n* = 15).

Items	Groups			*SEM*	*P*-value		
	CON group	AM group	FAM group		Groups	Time	Groups × Time
Body weight of male lambs (*n* = 8)/kg	9.59^Bb^	10.58^ABa^	11.04^Aa^	0.40	0.01	< 0.01	< 0.01
Daily weight gain of male lambs (*n* = 8)/kg	0.20^Bb^	0.22^ABa^	0.23^Aa^	0.01	0.01	< 0.01	0.12
Body weight of female lambs (*n* = 7)/kg	9.07^Bb^	10.17^ABa^	10.64^Aa^	0.46	0.01	< 0.01	0.12
Daily weight gain of female lambs (*n* = 8)/kg	0.20^Bb^	0.23^ABa^	0.24^Aa^	0.01	0.01	< 0.01	0.92

Groups = main effect of treatment, Time = main effect of sampling day, Groups × Time = interaction between treatment and sampling day. The same applies to the table below. The letters indicate simple effect comparisons at specific time points. When no letter or the same letter appears in the data labels, the difference is not significant (*p* > 0.05); different lowercase letters indicate a significant difference (*p* < 0.05); and different uppercase letters indicate a highly significant difference (*p* < 0.01).

#### 3.2.2 Effects of supplementing ewes with Astragalus and fermented Astragalus on plasma GH and IGF-1 levels in lambs

As shown in Table 8, supplementation with Astragalus and fermented Astragalus significantly increased GH levels in lambs for both the treatment group and time effects, as well as the interaction between treatment and time (*p* < 0.01). Plasma IGF-1 levels were significantly higher in the AM and FAM groups than in the CON group for both the treatment group and time effects (*p* < 0.01); however, the treatment group and time interaction effect had no significant impact on plasma IGF-1 levels (*p* > 0.05).

**Table 8:** Effects of supplementing ewes with Astragalus and fermented Astragalus on plasma GH and IGF-1 levels in lambs (*n* = 6).

Items	Groups			*SEM*	*P*-value		
	CON group	AM group	FAM group		Groups	Time	Groups × Time
GH (pg/ml)	2.09^Cc^	2.91^Bb^	3.44^Aa^	0.10	< 0.01	0.01	< 0.01
IGF-1 (ng/ml)	99.13^Cc^	107.29^Bb^	116.58^Aa^	4.75	< 0.01	< 0.01	0.19

Groups = main effect of treatment, Time = main effect of sampling day, Groups × Time = interaction between treatment and sampling day. The same applies to the table below. The letters indicate simple effect comparisons at specific time points. When no letter or the same letter appears in the data labels, the difference is not significant (*p* > 0.05); different lowercase letters indicate a significant difference (*p* < 0.05); and different uppercase letters indicate a highly significant difference (*p* < 0.01).

#### 3.2.3 Effects of supplementing ewes with Astragalus and fermented Astragalus on plasma immunoglobulin levels in lambs

As shown in Table 9, supplementation with Astragalus and fermented Astragalus significantly increased the levels of plasma immunoglobulins IgA, IgG, and IgM in lambs (*p* < 0.01). In both the AM and FAM groups, IgA and IgG levels were significantly higher than those in the control group as the duration of supplementation increased (*p* < 0.01); plasma IgM levels in all three groups of lambs showed significant changes over time (*p* < 0.01).

**Table 9:** Effect of supplementing ewes with Astragalus and fermented Astragalus on plasma immunoglobulin levels in lambs (*n* = 6).

Items	Groups			*SEM*	*P*-value		
	CON group	AM group	FAM group		Groups	Time	Groups × Time
IgA (g/L)	3.60^B^	3.89^A^	3.98^A^	0.07	< 0.01	< 0.01	< 0.01
IgG (g/L)	12.42^B^	13.35^A^	13.83^A^	0.54	< 0.01	< 0.01	< 0.01
IgM (g/L)	3.94	4.30	4.24	0.09	0.11	< 0.01	0.01

Groups = main effect of treatment, Time = main effect of sampling day, Groups × Time = interaction between treatment and sampling day. The letters indicate simple effect comparisons at specific time points. When no letter or the same letter appears in the data labels, the difference is not significant (*p* > 0.05); and different uppercase letters indicate a highly significant difference (*p* < 0.01).

#### 3.2.4 Effects of supplementing ewes with Astragalus and fermented Astragalus on the antioxidant capacity of lamb plasma

As shown in Table 10, supplementation with fermented Astragalus significantly increased plasma CAT activity (*p* < 0.01) and decreased NO content (*p* < 0.01), but had no significant effect on T-AOC, SOD, GSH-Px, or MDA levels (*p* > 0.05).

**Table 10:** Effects of supplementing ewes with Astragalus and fermented Astragalus on the antioxidant capacity of lamb plasma (*n* = 6).

Items	Groups			*SEM*	*P*-value		
	CON group	AM group	FAM group		Groups	Time	Groups × Time
T-AOC (U/mL)	9.84	9.57	9.70	0.06	0.16	0.07	0.36
CAT (U/mL)	4.94^Cc^	5.52^Bb^	6.30^Aa^	0.15	< 0.01	< 0.01	0.01
SOD (U/mL)	12.25	13.87	14.89	0.65	0.18	< 0.01	0.12
GSH-Px (U/mL)	40.30	40.36	41.42	1.08	0.49	< 0.01	0.29
NO (nmol/mL)	165.09^Aa^	156.37^Bb^	149.62^Bb^	2.47	< 0.01	< 0.01	0.01
MDA (nmol/mL)	1.04	1.02	1.05	0.03	0.93	0.03	0.28

Groups = main effect of treatment, Time = main effect of sampling day, Groups × Time = interaction between treatment and sampling day. The letters indicate simple effect comparisons at specific time points. In the data tables, the absence of a letter or the presence of the same letter in the superscript indicates no significant difference (*p* > 0.05); different lowercase letters indicate a significant difference (*p* < 0.05); and different uppercase letters indicate a highly significant difference (*p* < 0.01).

## 4 Discussion

As the principal digestive organ in ruminants, the rumen hosts an extensive and intricate microbial ecosystem, which is essential for modulating host physiology and mediating digestive metabolism (Stewart et al., 2019). These microbial populations are pivotal not only for fiber degradation and nutrient metabolism but also for host health, as they maintain a dynamic equilibrium in community structure and relative abundance (Yong et al., 2025). Supplementation with fermented Astragalus combined with beneficial bacteria confers dual benefits to animals, including enhanced growth performance, immune function, antioxidant capacity, and anti-inflammatory response, as well as improved disease resistance through inhibition of pathogenic bacteria and potentiation of immune responses (Abd El-Hack et al., 2020; Shi, 2024; Han et al., 2025; Li S. et al., 2025). Under the conditions of this trial, *Bacteroidota, Bacillota*, and *Methanobacteriota* were the dominant microbial phyla across all groups. The AM group exhibited a higher relative abundance of *Bacteroidota*, whereas the FAM group showed higher relative abundances of *Bacillota* and *Methanobacteriota*. Research indicates that *Bacteroidota* are highly efficient in carbohydrate degradation, including the breakdown of cellulose, hemicellulose, pectin, and resistant starches present in plant-based feeds. These phyla are equipped with an array of cell-surface glycoside hydrolases, polysaccharide-depolymerizing enzymes, and associated enzymatic systems that sequentially degrade complex carbohydrates to low-molecular-weight products, including monosaccharides and oligosaccharides (Edwards et al., 2010). These breakdown products provide carbon and energy for microbial growth and supply essential nutrients to other rumen microbes, supporting a stable microenvironment and efficient nutrient conversion (Li et al., 2021). The *Bacteroidota* possesses carbohydrate-degrading capabilities, enabling it to specifically recognize and cleave the glycosidic bonds of Astragalus polysaccharide molecules, thereby gradually breaking down the macromolecular polysaccharides into small-molecule sugars that can be absorbed and utilized (Nakamura et al., 2023). He et al. (2022) noted that Bacillota is positively associated with gut health, as this phylum contains a large number of butyrate-producing bacteria and plays a significant role in energy metabolism and anti-inflammation. Within the FAM group, both the *Bacillota* and *Methanobacteriota* exhibit high relative abundances. The *Bacillota* promotes the production of gut metabolites such as butyric acid, helps regulate gut microbiota balance, suppresses inflammatory responses, and strengthens the intestinal barrier. The results of the present study are consistent with these findings. The increase in the relative abundance of the *Methanobacteria* may be related to the regulation of hydrogen metabolism in the gut (Borrel et al., 2023). Villanueva-Millan et al. (2022) reported that Methanobacteriota can utilize hydrogen produced by the gut microbiota and convert it to methane. This process reduces hydrogen accumulation and optimizes the intestinal metabolic environment, thereby indirectly influencing the host's energy metabolism. Additionally, *Bacillus subtilis* present in fermented Astragalus promotes intestinal development, increases intestinal villus height, and produces beneficial metabolites, including butyric acid (Wang et al., 2011). Additionally, the FAM group exhibited relatively high abundances of *Spirochaetota* and *Actinobacteriota*. Polkade et al. (2016) noted that the *Actinobacteria* phylum can degrade cellulose and secrete various bioactive compounds, including enzymes, antimicrobial substances, and antibiotics, while also harboring a variety of probiotics. Tokuda et al. (2018) found that the *Spirochaetota* is involved in fiber degradation, and that its abundance varies in response to changes in fermentation substrates and enhancements in overall gut microbiome function.

Fermentation by the rumen microbiota generates metabolites such as short-chain fatty acids and amino acids, which are absorbed and metabolized by the liver via the portal vein before entering the peripheral circulation. This process establishes a characteristic plasma metabolic profile that mediates the host's energy metabolism and immune regulation (Xue et al., 2020). In this trial, plasma differential metabolites were detected and screened on the day of lambing. Comparisons between the FAM and CON groups, as well as between the FAM and AM groups, revealed that plasma differential metabolites were primarily concentrated in the tryptophan metabolic pathway, whereas those between the FAM and CON groups were mainly enriched in the alpha-linolenic acid metabolic pathway. It is hypothesized that plasma differential metabolites following Astragalus supplementation are primarily metabolized through the tryptophan pathway. Notably, fermentation of Astragalus with *Bacillus subtilis* and *Bacillus licheniformis* modifies its bioactive constituents, consequently impacting the metabolic pathways of the plasma differential metabolites. We speculate that plasma differential metabolites following Astragalus supplementation are primarily metabolized via the tryptophan pathway, whereas fermentation by Bacillus subtilis and Bacillus licheniformis alters the active components of Astragalus, thereby modulating the metabolic pathways of these differential metabolites. Tryptophan is primarily metabolized through the serotonin pathway and the kynurenine pathway (KP), generating multiple bioactive metabolites such as serotonin and kynurenine (Song et al., 2017; Kiluk et al., 2021). Zhao X. et al. (2024) noted that serotonin is a key neurotransmitter regulating physiological processes such as feeding, mood, and sleep in animals. Its metabolite, melatonin, is closely associated with the estrous cycle, reproductive performance, and antioxidant function in animals (Song et al., 2016). The levels of quinolinic acid and picolinic acid, which are metabolites of tryptophan, were significantly upregulated. Zhang et al. (2024) noted that quinolinic acid is a tryptophan metabolite and that its accumulation can induce oxidative stress. The levels of quinolinic acid and picolinic acid, both tryptophan metabolites, were significantly upregulated. However, quinolinic acid was not detected in the FAM group. Therefore, we hypothesize that fermented Astragalus more effectively inhibits the conversion of tryptophan metabolism toward quinolinic acid production, thereby reducing the risk of oxidative stress. The significant upregulation of metabolites in the tryptophan metabolic pathway observed in this study was likely attributable to the actions of Astragalus polysaccharides and flavonoids. Exhibiting anti-inflammatory, antioxidant, and immunomodulatory activities, these two classes of bioactive compounds reduce pro-inflammatory cytokines, stabilize neuroendocrine regulation, and alleviate stress responses, thus promoting favorable conditions for parturition and postpartum recovery.

On the other hand, the plasma metabolites that differed between the fermented Astragalus group and the control group were primarily concentrated in the alpha-linolenic acid metabolic pathway. Following supplementation with fermented Astragalus, the levels of metabolites such as medicarpin and 3-hydroxyhexadecanoylcarnitine were downregulated. Medicarpin is a flavonoid compound, and its significant decrease may be attributed to the microbial conversion of flavonoid glycosides present in Astragalus root during the fermentation process (Ma et al., 2025). It is hypothesized that medicarpin, through its anti-inflammatory and immune-enhancing effects, may improve the body's ability to resist pathogens and regulate the intensity of inflammatory responses (Hassan et al., 2025). During mitochondrial β-oxidation of hexadecanoic acid (palmitic acid), incomplete shortening of the carbon chain or altered activity of specific enzymes leads to the production and release of the intermediate metabolite 3-hydroxyhexadecanoylcarnitine into the bloodstream (Fu et al., 2021). In addition, supplementation with fermented Astragalus significantly increased plasma levels of the glycoconjugated bile acids glycocholic acid, glycodeoxycholic acid, and glycolithocholic acid in ewes. The secondary conjugated bile acids glycodeoxycholic acid and glycolithocholic acid are derived from primary bile acids through the sequential actions of microbial deconjugation and 7α-dehydroxylation, followed by reconjugation with glycine in the liver. These acids are involved not only in fat digestion and absorption but also serve as signaling molecules that modulate carbohydrate and lipid metabolism, energy balance, and immune regulation (Meessen et al., 2024). Although Astragalus polysaccharides significantly downregulate hepatic bile acid synthase expression (Zheng et al., 2024), plasma glycine-conjugated bile acids were markedly elevated in FAM ewes on the day of parturition, presumably due to the ability of fermented Astragalus to improve intestinal barrier function and increase enterohepatic circulation efficiency of bile acids. Additionally, fermented Astragalus polysaccharides may reduce the degradation and excretion of bile acids in the intestine by modulating gut microbiota composition, thereby increasing their accumulation in plasma. Ultimately, this improves fat digestion and absorption capacity during parturition, ensuring an adequate energy supply. In the FAM group, homo-γ-linolenic acid (homo-γ-LA) was significantly downregulated and was primarily enriched in the linolenic acid metabolic pathway. Homo-γ-linolenic acid is a precursor of anti-inflammatory prostaglandins; *in vivo*, it is metabolized by cyclooxygenase (COX) to produce a series of prostaglandins (PGE1), which exert vasodilatory, antiplatelet, and anti-inflammatory effects. Gallagher (2016). Supplementation with fermented Astragalus increases serum T-AOC and SOD activity and enriches metabolites associated with antioxidant and growth-promoting effects (Wang et al., 2024). cis-γ-Linolenic acid, a polyunsaturated fatty acid, is highly prone to oxidative stress during its synthesis. Fermented Astragalus contains active components (e.g., polysaccharides and glycosides) that exert anti-inflammatory and antioxidant effects, thereby regulating arachidonic acid and PGE1 synthesis and metabolism, and alleviating oxidative stress and inflammation in parturient ewes.

During the final stage of pregnancy, fetal growth accelerates and body weight increases rapidly; consequently, the mother must supply additional nutrients. Failure to provide adequate nutrients at this stage may impair fetal development, resulting in lambs with low birth weight, poor body condition, and compromised immune function, which in turn increases mortality rates (Yang, 2020). Breast milk, particularly colostrum, is of extremely high value and represents the sole source of passive immunity for newborn lambs. Its immunoglobulin, fat, and protein contents are critical determinants of the lambs' early immune protection and subsequent growth potential (Hamer et al., 2023). Studies have shown that dietary supplementation with an appropriate amount of Astragalus enhances the immune and antioxidant capacities of animals (Han et al., 2022). Zhong et al. (2012) reported that Astragalus can increase plasma SOD activity. A study by Shen et al. (2014) reported that Astragalus enhances antioxidant capacity and alleviates oxidative stress in dairy cows. Furthermore, dietary supplementation with Astragalus or its fermented form during the experimental period has been shown to increase milk yield (Li et al., 2012; Du et al., 2013; Lian, 2015). In this experiment, dietary supplementation with Astragalus or fermented Astragalus significantly improved colostral protein and lactose contents, and tended to increase milk yield and antioxidant capacity on the day of parturition. It is hypothesized that supplemental feeding of Astragalus or fermented Astragalus to Turpan Black sheep enhances maternal resistance, thereby facilitating the transfer of immunologically active substances across the placental barrier to the fetuses, and ultimately providing lambs with high-quality colostrum. This established a foundation for lambs to acquire passive immunity at birth and to develop subsequent immune function and antioxidant capacity. As the primary immunologically active component in colostrum, IgG plays a crucial role in the development of the gastrointestinal immune system in young animals (Li X. et al., 2025; Cheng, 2023). In ruminants, IgG constitutes the majority of immunoglobulin activity, and its levels were significantly elevated in lambs from both the AM and FAM groups. Furthermore, Astragalus and fermented Astragalus may enhance the synthesis of milk protein and lactose by regulating the metabolic function of the maternal mammary tissue and promoting the absorption and conversion of nutrients by mammary epithelial cells. Milk protein is an essential component of lamb tissues and immunoglobulins; increasing its content enhances the nutritional value of colostrum and provides newborn lambs with an adequate supply of amino acids (Wang et al., 2022). As the primary carbohydrate in colostrum, lactose not only provides energy for lambs but also promotes the colonization and proliferation of beneficial gut microbiota, thereby improving gut health and indirectly enhancing digestive capacity and immunity in the young (Gao et al., 2024). The increased milk yield ensured adequate milk intake for newborn lambs, preventing hunger-induced growth retardation, consistent with earlier reports. Beyond confirming the beneficial effects of Astragalus and fermented Astragalus on milk quality, yield, and antioxidant capacity in ewes, this study further suggests that such supplementation may affect lamb body weight and plasma hormone levels, either transplacentally or via milk. Active components, such as polysaccharides, in Astragalus and fermented Astragalus can promote lactation and improve milk quality in ewes (Chen, 2022), thereby increasing the average daily gain of lambs, stimulating the secretion of hormones such as GH and IGF-1 (Liu et al., 2025), and enhancing antioxidant capacity and immune function (Wang and Wu, 2023). Studies have shown that Astragalus polysaccharides promote cell proliferation and differentiation (Su et al., 2023), which may directly influence the development of fetal tissues, including muscle and bone, thereby providing a physiological basis for increased lamb birth weight.

**Figure 8:**
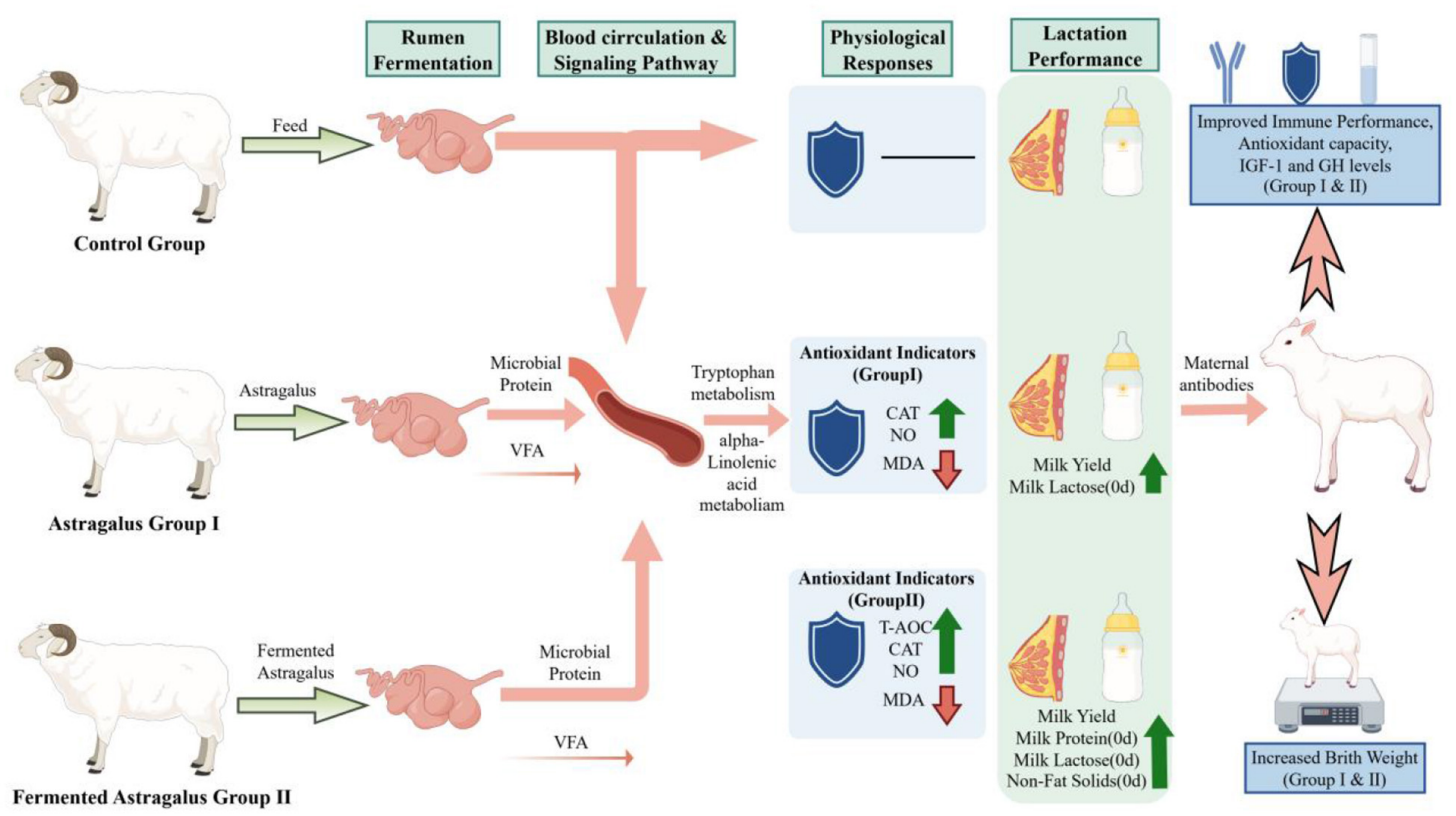
Supplementation of fermented Astragalus in ewes: effects on rumen microbiota structure, plasma metabolites, antioxidant status, and offspring immune function and growth performance. Control Group, CON group; Astragalus groupI, AM group; fermented Astragalus group II, FAM group.

In summary, the results of this experiment indicate that both Astragalus and fermented Astragalus can improve the reproductive performance of ewes during late pregnancy (Figure 8). Both supplements enhance maternal immunity and antioxidant capacity, promote mammary gland metabolism and colostrum synthesis, and improve nutrient conversion efficiency by optimizing the rumen microbial flora. Metabolomic analysis revealed that Astragalus primarily regulates the tryptophan metabolic pathway, whereas fermented Astragalus mainly affects α-linolenic acid and bile acid metabolism. Furthermore, fermented Astragalus showed superior efficacy in inhibiting quinolinic acid formation and promoting the digestion and absorption of dietary fat, reflecting the synergistic effects of microbial fermentation. Astragalus and its fermented form may exert positive effects on lamb growth performance, antioxidant capacity, and immune function either through the placental barrier or via milk; however, the specific mechanisms require further investigation.

## 5 Conclusion

Under the conditions of this experiment, Astragalus supplementation significantly increased the relative abundance of the *Bacteroidetes*. Plasma levels of melatonin, picolinic acid, and other differential metabolites were highly significantly upregulated and were significantly enriched in the tryptophan metabolic pathway. On the day of parturition, plasma CAT activity in ewes increased highly significantly, which not only enhanced milk yield but also improved milk composition. In lambs, IgG, IgM, and CAT activity all showed highly significant increases. Plasma GH and IGF-1 levels were higher than those in the control group, thereby promoting lamb growth performance.

Following supplementation with fermented Astragalus, the relative abundance of *Bacteroidetes* in the rumen of ewes decreased, whereas those of *Methanobacteria* and *Spirochaetes* increased significantly. Plasma metabolites, including 3-hydroxyhexadecanoylcarnitine and cis-γ-linolenic acid, were highly significantly downregulated and were enriched in the α-linolenic acid metabolic pathway. Fermented Astragalus moderately increased milk yield and improved milk composition, including protein and lactose content. On the day of parturition, ewes showed highly significant increases in CAT and T-AOC, as well as in plasma IgA and IgG proportions. In lambs, growth hormone (GH) and IGF-1 levels were highly significantly elevated, and growth performance was significantly improved.

In comparison, fermented Astragalus demonstrated superior effects to raw Astragalus.

### 5.1 Limitations

Our study monitored lambs only until 45 days of age (pre-weaning). Therefore, we cannot determine whether the observed improvements in IgG levels and growth performance persist beyond weaning or extend into later productive stages (e.g., post-weaning growth, feedlot performance, or reproductive lifetime). It is possible that early-life benefits diminish after weaning due to changes in diet, environment, or immune challenges. Alternatively, there may be long-lasting programming effects. Future longitudinal studies with extended follow-up are required to address this important question.

It should be noted that this study was based on a specific basal diet under feedlot conditions. The response to Astragalus may differ under grazing systems, where animals consume diverse forages and experience different levels of physical activity and immune challenges. Therefore, further investigations are warranted to compare the efficacy of Astragalus across contrasting feeding management systems before extrapolating our conclusions to broader production scenarios.

This study was limited by the use of a single supplementation dose (2 g/day). Future dose-response studies are needed to establish the optimal dosage for practical application.

## Data Availability

The raw data generated in this study can be found in the NCBI Sequence Read Archive (SRA), accession PRJNA1423633: https://www.ncbi.nlm.nih.gov/sra/PRJNA1423633.
