# Supplementary material for: The effects of supplementing Astragalus and fermented Astragalus on lactation performance, rumen microbiota, and lamb weight gain in Turpan black sheep
Source: Front Microbiol. 2026 May 22;17:1810954. doi: 10.3389/fmicb.2026.1810954 (PMC13236909; doi:10.3389/fmicb.2026.1810954)
Supplement: Supplementary file 1 [file Data_Sheet_1.docx]

**Appendix**

**1.1Data availability statement**

The datasets presented in this study can be found in online repositories. NCBI：<https://www.ncbi.nlm.nih.gov/sra/PRJNA1423633>. The names of the repository/repositories and accession number(s) can be found in the article/[Supplementary material](#Supplementary).

**1.2** **Effects of Supplementing with Astragalus and Fermented Astragalus on the Composition of the Rumen Microbiota in Ewes**

Table 1 Effects of Supplementing with Astragalus and Fermented Astragalus on the Phylum Composition of the Rumen Microbiota in Ewes

| Item | Groups | | | *P*-value |
| --- | --- | --- | --- | --- |
|  | CON group | AM group | FAM group |  |
| Bacteroidota | 54.03±10.22^a^ | 54.16±6.73^a^ | 41.43±11.41^b^ | 0.05 |
| Bacillota | 34.09±6.25 | 35.45±3.54 | 39.85±7.19 | 0.24 |
| Methanobacteriota | 7.76±4.85^b^ | 6.79±5.95^b^ | 14.99±5.37^a^ | 0.04 |
| Patescibacteria | 1.86±1.76 | 1.83±0.80 | 0.91±0.50 | 0.30 |
| Spirochaetota | 1.12±0.61^ab^ | 0.67±0.37^b^ | 1.60±0.63^a^ | 0.04 |
| Actinomycetota | 0.33±0.26 | 0.46±0.17 | 0.62±0.30 | 0.17 |
| Fibrobacterota | 0.36±0.40 | 0.14±0.15 | 0.28±0.27 | 0.47 |
| Thermodesulfobacteriota | 0.26±0.14 | 0.33±0.19 | 0.15±0.14 | 0.19 |
| Pseudomonadota | 0.10±0.14 | 0.05±0.06 | 0.06±0.06 | 0.59 |
| Cyanobacteriota | 0.01±0.02 | 0.02±0.03 | 0.05±0.06 | 0.43 |

Note: In the data tables, the absence of a letter or the presence of the same letter in the superscript indicates no significant difference (*P* >0.05); different lowercase letters indicate a significant difference (*P* <0.05).

Table 2 Effects of Supplementing with Astragalus and Fermented Astragalus on the Family Composition of the Rumen Microbiota in Ewes

| Item | Groups | | | *P*-value |
| --- | --- | --- | --- | --- |
|  | CON group | AM group | FAM group |  |
| Prevotellaceae | 20.21±9.97 | 23.54±12.48 | 14.68±7.68 | 0.34 |
| F082 | 12.06±4.29 | 12.80±6.26 | 9.74±2.56 | 0.51 |
| Methanobacteriaceae | 7.76±4.85^b^ | 6.79±5.95^b^ | 14.99±5.37^a^ | 0.04 |
| Rikenellaceae | 13.92±2.32 | 11.24±2.53 | 12.59±3.10 | 0.25 |
| Oscillospiraceae | 7.99±2.20 | 9.37±3.59 | 10.22±3.17 | 0.46 |
| Lachnospiraceae | 8.51±2.38 | 9.02±2.39 | 8.57±3.30 | 0.94 |
| Muribaculaceae | 4.73±3.89 | 4.70±1.92 | 2.31±0.53 | 0.20 |
| Christensenellaceae | 5.59±3.64 | 6.13±2.44 | 8.10±3.13 | 0.36 |
| Ruminococcaceae | 2.39±1.00^ab^ | 1.95±0.24^b^ | 4.68±3.21^a^ | 0.05 |
| Bacteroidales_RF16_group | 1.83±3.26 | 0.61±0.27 | 0.95±0.63 | 0.54 |

Note: In the data tables, the absence of a letter or the presence of the same letter in the superscript indicates no significant difference (*P* >0.05); different lowercase letters indicate a significant difference (*P* <0.05).

Table 3 Effects of Supplementing with Astragalus and Fermented Astragalus on the Genus Composition of the Rumen Microbiota in Ewes

| Item | Groups | | | P-value |
| --- | --- | --- | --- | --- |
|  | CON group | AM group | FAM group |  |
| *Xylanibacter* | 12.72±8.95 | 14.88±7.91 | 9.80±6.68 | 0.55 |
| *F082*  *unclassified_F082* | 12.06±4.29 | 12.80±6.26 | 9.74±2.56 | 0.51 |
| *Methanobrevibacter* | 7.39±4.87^b^ | 6.65±5.94^b^ | 14.69±5.24^a^ | 0.39 |
| *Rikenellaceae_RC9_gut_group* | 13.30±2.14 | 10.69±2.22 | 12.07±2.94 | 0.22 |
| *unclassified_Muribaculaceae* | 4.73±3.89 | 4.70±1.92 | 2.31±0.53 | 0.20 |
| *Christensenellaceae_R-7_group* | 4.99±3.43 | 5.11±1.46 | 7.33±2.86 | 0.28 |
| *NK4A214_group* | 3.43±1.74 | 4.88±3.07 | 6.44±2.76 | 0.17 |
| *Ruminococcus* | 1.82±1.01^ab^ | 1.48±0.22^b^ | 4.25±3.27^a^ | 0.05 |
| *unclassified_Bacteroidales_RF16_group* | 1.83±3.26 | 0.61±0.27 | 0.95±0.63 | 0.54 |
| *Succiniclasticum* | 3.89±1.95 | 2.95±1.56 | 3.19±2.04 | 0.64 |

Note: In the data tables, the absence of a letter or the presence of the same letter in the superscript indicates no significant difference (*P* >0.05); different lowercase letters indicate a significant difference (*P* <0.05).
